# Signaling Pathways Tuning Innate Lymphoid Cell Response to Hepatocellular Carcinoma

**DOI:** 10.3389/fimmu.2022.846923

**Published:** 2022-02-23

**Authors:** Elsa Bourayou, Rachel Golub

**Affiliations:** Institut Pasteur, Université de Paris, INSERM U1223, Lymphocyte and Immunity Unit, Paris, France

**Keywords:** HCC, NASH, NK cells, ILC1, liver, inflammation, Notch and TGF-β pathways

## Abstract

Hepatocellular carcinoma (HCC) is one of the deadliest cancers worldwide and its incidence continues to rise globally. Various causes can lead to its development such as chronic viral infections causing hepatitis, cirrhosis or nonalcoholic steatohepatitis (NASH). The contribution of immune cells to HCC development and progression has been extensively studied when it comes to adaptive lymphocytes or myeloid populations. However, the role of the innate lymphoid cells (ILCs) is still not well defined. ILCs are a family of lymphocytes comprising five subsets including circulating Natural Killer (NK) cells, ILC1s, ILC2s, ILC3s and lymphocytes tissue-inducer cells (LTi). Mostly located at epithelial surfaces, tissue-resident ILCs and NK cells can rapidly react to environmental changes to mount appropriate immune responses. Here, we provide an overview of their roles and actions in HCC with an emphasis on the importance of diverse signaling pathways (Notch, TGF-β, Wnt/β-catenin…) in the tuning of their response to HCC.

## Introduction

Hepatocellular carcinoma (HCC) accounts for up to 90% of all cases of primary liver cancers while the intrahepatic cholangiocarcinoma represents roughly 10% (1, 2). HCC is one of the deadliest cancers worldwide and its incidence continues to rise globally (3). Various causes can lead to the development of an HCC. Chronic viral infections by hepatitis B virus (HBV) or hepatitis C virus (HCV) still account for more than half its cases (4). However, antiviral therapies against HCV have considerably been improved over the last decades and HCV clearance is effective is most patients (5). HBV infection remains for life but can be prevented by vaccination (6). On the other hand, cirrhosis is the highest risk for progression towards HCC with 40% of the patients developing liver cancer. Cirrhosis can be alcohol-related or can result from metabolic syndrome-associated non-alcoholic steatohepatitis (NASH). The latter is thought to become the most leading cause of HCC and is already the fastest growing aetiology in western countries where the unbalanced food diets are responsible for a rise in obesity (7).

HCC is frequently diagnosed at later stages and there is no curative therapy for an advanced HCC. Thus, the early diagnosis is one of the biggest challenge (8). Over the past few years, the early diagnosis of HCC has relied on surveillance with ultrasonography (US) and serological assessments of alpha-fetoprotein (AFP). However, the specificity and sensitivity of US/AFP is not satisfactory enough to detect early onset HCC. HCC is mostly treated with surgical resection and liver transplantation (9, 10). Systemic therapies targeting the immune system have emerged and offer an alternative to the conventional therapies (11). Thus, it remains important to continue improving our knowledge of the immune mechanisms in the context of HCC.

The liver is an organ that contains a large number of immune cells. The contribution of T and B lymphocytes to HCC (12–14) as well as that of myeloid populations (15–18) have been extensively studied but less is known about the involvement of innate lymphoid cells (ILCs) in HCC. ILCs are the innate counterpart of T lymphocytes but lack antigen receptors. They are divided into five subsets based on their developmental pathway, phenotype and function: NK cells, helper ILC1s, ILC2s and ILC3s that mirror Th1, Th2 and Th17 lymphocytes, and the lymphoid tissue-inducer (LTi) cells. Canonical signaling pathways such as Notch, Wnt/β-catenin and TGF-β pathways, were shown to be involved in ILC differentiation and functional activation. Thus, in addition to discussing the roles of ILCs in the HCC context, this review aims at highlighting how these signaling pathways might impact the functions of ILCs in liver cancer and what therapeutic strategies could be considered.

## Presentation Of ILC Subsets

**Figure 1:**
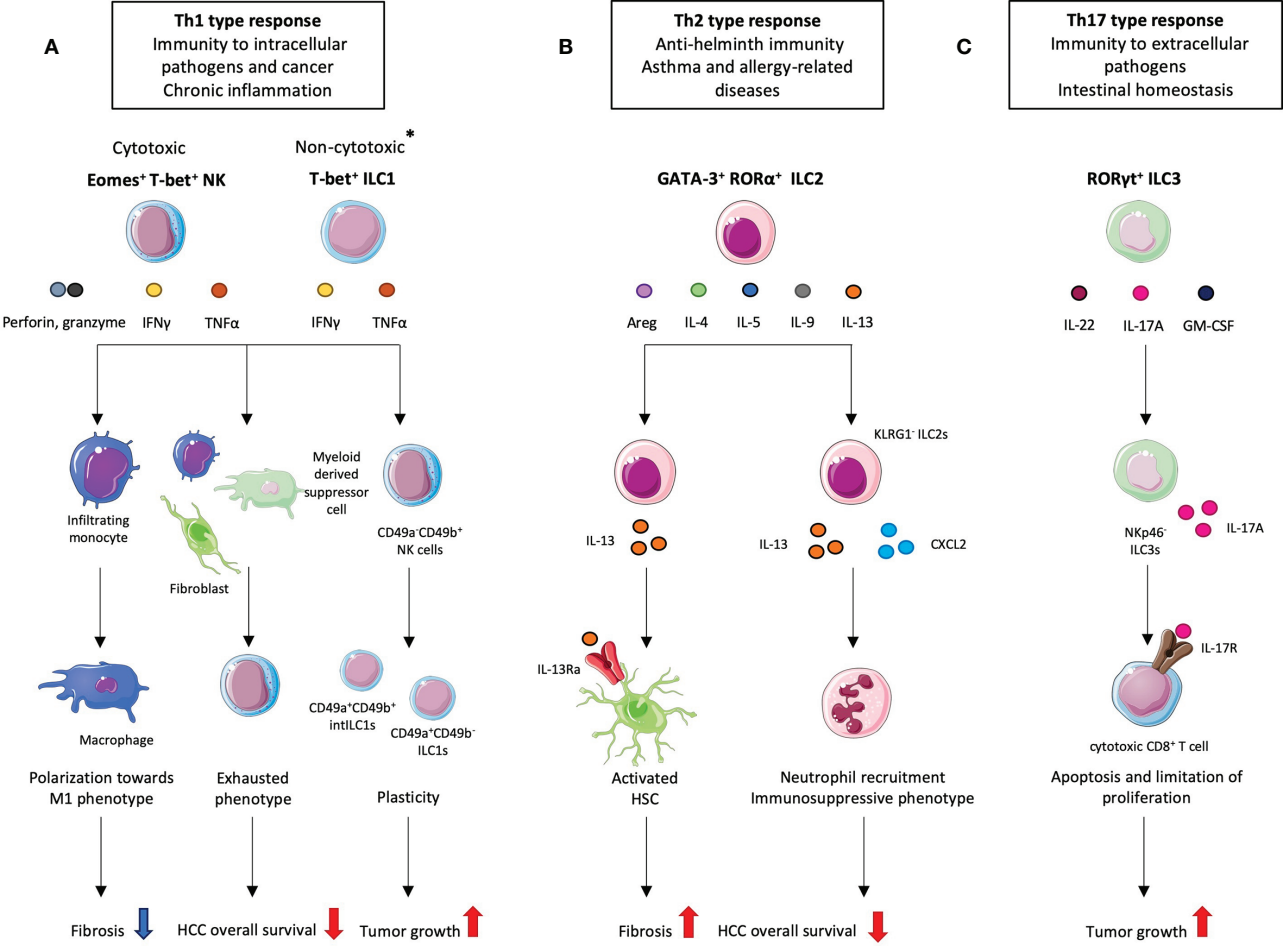
Different ILC subsets and their pro- and anti-tumoral roles in hepatocellular carcinoma (HCC). ^*^ Most ILC1 subsets are non-cytotoxic however hepatic ILC1s can secrete perforin and granzyme B. **(A)** Given their cytotoxic activity, liver NK cells and liver ILC1s appear as good candidates in the anti-tumor response. Indeed, at the fibrosis stage, which precedes HCC development, NK cells exhibit a protective role by promoting macrophages towards an M1 phenotype. However, in HCC, NK cells display an exhausted phenotype resulting from interaction with cancer-associated fibroblasts and/or myeloid-derived suppressor cells which correlates with decreased patient overall survival. NK cells also convert to intermediate (int) ILC1s. **(B)** ILC2s are implicated at the fibrotic stage where they play a detrimental role. IL-33-activated ILC2s produce IL-13 which activates hepatic stellate cells (HSCs) that start secreting collagen. At the HCC stage, ILC2s tend to promote its progression by recruiting neutrophils. This effect is mediated by the loss of KLRG1 and increased secretion of CXCL2 and IL-13. **(C)** NKp46^-^ ILC3s have been shown to promote HCC through the IL-23/IL-17A axis. ILC3-produced IL-17A directly inhibits CD8^+^ T cell proliferation and cytotoxic activity which subsequently lead to tumor growth.

The current ILC nomenclature is based on their phenotype and functions (19, 20). ILC1s and NK cells share a lot of similarities with Th1 lymphocytes. They both produce type 1 cytokines such as interferon gamma (IFNγ) and tumor necrosis factor alpha (TNFα) upon activation by interleukin (IL)-12, IL-15 and/or IL-18 (**Figure 1A**). They are involved in immunity to intracellular bacteria and viruses and play a protective role in cancer. They both require the expression of the T-box transcription factor T-bet however, NK cells also rely on eomesodermin (Eomes) expression for development and function (21) (**Figure 1A**). Contrary to most ILC1 subsets, liver ILC1s can exhibit cytotoxic activities like NK cells with the expression of TNF-related apoptosis-inducing ligand (TRAIL) as well as granzyme b and perforin (22). ILC2s mirror Th2 lymphocytes. They depend on the expression of the transcription factors GATA-3 and RORα (23, 24) and secrete type 2 cytokines such as IL-4, IL-5, IL-9, IL-13 and amphiregulin (Areg) (25, 26) (**Figure 1B**). Their activation is dependent on the cytokines IL-33, IL-25 and on the thymic stromal lymphopoeitin (TSLP). They are essential in the anti-helminth immune response but were shown to be detrimental in asthma and allergy contexts. The role of ILC2s in cancer is an emergent field of study with pro-tumor and anti-tumor actions depending on the context (27, 28). Adult ILC3s are enriched in the intestine, rely on the expression of retinoic acid related orphan receptor (ROR)γt encoded by the *RORc* gene and are activated by IL-23 and IL-1β. They produce Th17-related cytokines namely IL-17A and IL-22 as well as GM-CSF (**Figure 1C**). ILC3s are further divided into three subsets: one that expresses the natural cytotoxicity receptor (NCR), first discovered on NK cells and ILC1s, and the other one that does not. NCR^+^ ILC3s arise from the NCR^-^ ILC3s and mainly produce IL-22 while the NCR^-^ subset produces high amounts of IL-17A (29, 30). ILC3s are involved in the defense against extracellular pathogens and in intestinal homeostasis but were also shown to play a role in chronic intestinal inflammation (31, 32). Patients suffering from Inflammatory bowel disease (IBD) are at high risk of developing cancer (33) and ILC3s are known to be central players in the aggravation of IBD (34) which suggests that these cells can also be involved in cancer progression.

Under specific circumstances, plasticity between the different subsets was demonstrated in many studies. Cellular plasticity was first described by Helen Blau in 1985 (35). In her studies, muscle cell identity was regulated by extracellular factors. Cell identity was no longer fixed but subjected to regulation by environmental cues. In mice, CCR6^-^ RORγt^+^ ILC3s can downregulate their expression of *Rorc* to the benefit of *Tbx21* coding for T-bet during *Salmonella enterica* infection. These “ex-ILC3s” exhibit ILC1-like properties with a T-bet-driven production of IFNγ that contributes to the protection of the epithelial barrier during the infection (36). This conversion can also be dependent on extrinsic signals such as the production of IL-12 by dendritic cells and monocytes (37–39). Interestingly, the opposite plasticity was shown as possible with an IL-23- and IL-1β-mediated *in vitro* transdifferentiation of human ILC1s into ILC3-like cells (39). ILC2s also displayed plastic properties in several studies. In response to IL-12, human peripheral blood ILC2s can upregulate T-bet expression and start producing IFNγ both *in vitro* and *in vivo* (40–42). In some studies, the expression of GATA-3 and Type 2 cytokine production were maintained giving a mixed ILC1/ILC2 phenotype to the cells (40, 42). In another one, the Th2-related pathway was downregulated in human ILC2s following *in vitro* culture with IL-1β and IL-12 (41). Interestingly, *in vitro* culture of human ILC2s with IL-4 was able to prevent such conversion indicating its crucial role in maintaining the ILC2 identity (41). The importance of these two cytokines in ILC2 plasticity was corroborated by the fact that ILC2 frequency is decreased in tissues from patients with severe chronic obstructive pulmonary disease (COPD) displaying an IL-12 signature while patients with chronic rhinosinusitis with nasal polyps (CRSwNP) displaying elevated eosinophil-derived IL-4 showed accumulation of ILC2s (41).

In contrast to NK cells that can circulate throughout the body, the other ILC subsets are mostly tissue-resident and are enriched at the barrier surfaces where they maintain tissue integrity in the steady-state. Thus, ILCs are considered as important players in the immune response that can rapidly react to tissue disruption or infection by the secretion of cytokines.

## Pro- And Anti-Tumoral Roles OF ILCs in HCC

### NK and ILC1s

In the liver, NK and ILC1s represent 30-50% of total lymphocytes in humans and around 10% in mice. Murine liver NK cells display a CD49a^-^ CD49b^+^ T-bet^+^ Eomes^+^ phenotype. They are located in the sinusoids and can recirculate. Mouse liver ILC1s are characterized as CD49a^+^ CD49b^-^ T-bet^+^ Eomes^-^ and are considered as liver-resident. In humans, liver NK cell population comprises CD56^dim^ and CD56^bright^ subsets. The CD56^bright^ population express high levels of Eomes and could thus be considered as conventional NK cells. However, they also express residency markers such as CXCR6 and CD69 (43–45). Human hepatic ILC1s were not successfully identified probably due to a lack of specific marker. Nonetheless, Marquardt et al. proved the existence of a unique subset of human liver CD56^bright^ NK cells expressing CD49a but not CD49b and whose lineage depends on T-bet but not on Eomes. After *in vitro* stimulation, these cells showed enhanced cytokine production but decreased degranulation capacity compared to CD49a^-^ NK cells (46). These results suggest that CD56^bright^ CD49a^+^ CD49b^-^ NK cells could be the human counterparts of mouse hepatic ILC1s.

In obesity-associated NASH mouse models, one of the most rising cause for HCC, NK cells have contrasting roles depending on the diet and the various parameters such as diet kinetics. In methionine and choline deficient diet (MCD)-induced NASH, NKp46^+^ NK1.1^+^ CD49b^+^ cell numbers are increased and were shown to prevent fibrosis by polarizing infiltrating monocytes towards M1 type macrophages in the liver (47, 48) (**Figure 1A**). This suggest a protective role for NK cells keeping the disease from progressing towards fibrosis and subsequently to HCC. Nonetheless, other studies showed a detrimental role for NK cells in the development of NASH. IL-15 knockout NK-deficient mice displayed an attenuated NASH in response to high fat diet (HFD) (49). Wang et al. showed that murine NK cells secrete higher levels of pro-inflammatory cytokines in NASH which subsequently activate hepatocytes through JAK/STAT and NF-kB signaling. This induces hepatocyte damage and apoptosis while NK cell depletion resulted in alleviation of the disease (50).

In HCC, the role of NK cells has been extensively studied given their cytotoxic capacities. The number of infiltrating CD56^+^ NK cells in the liver of patients suffering from HCC was positively correlated with the overall survival and cancer cell elimination suggesting a protective role (51, 52). However, decreased numbers of NK cells are observed in HCC patients (53, 54). NK cells also exhibit an exhausted phenotype with alteration of their cytotoxic activity and IFNγ production (53, 55). Several mechanisms are involved in the impairment of NK functions (**Figure 1A**). In one study, patients with severe HCC showed a correlation between the infiltration of monocytes to the tumor and NK cell exhaustion. Co-cultures of NK cells with these tumor-derived monocytes revealed that they can directly regulate NK cells through the CD48/2B4 (CD244) axis. Blockade of CD244 on NK cells prevented their dysfunction (55). In another study, myeloid-derived suppressor cells (MDSCs) present in the tumor of HCC patients were able to directly inhibit NK cells function through a cell-to-cell contact involving the cytotoxicity receptor NKp30 (15).

The role of ILC1s in HCC has been poorly studied. Contrary to most ILC1 subsets, mouse liver ILC1s express perforin and granzyme b. They are thus capable of lysing cells although they remain less efficient than NK cells (22). In a fibrosarcoma model where MCA1956 cell line was subcutaneously injected into mice, an intermediate CD49a^+^ CD49b^+^ population was detected in the tumor and named intermediate (int)ILC1s (56). These cells came from the conversion of NK cells into ILC1-like cells, a process which was mediated by TGF-β *in vitro* and *in vivo* (**Figure 1A**). While NK cells were able to control tumor growth and metastasis, ILC1s and intILC1s upregulated tumor progression and resistance-related pathways with a higher expression of inhibitory receptors (NKG2A, KLRG1) and the production of PDGF-AB, a pro-angiogenic molecule. Moreover, NK-derived intILC1s and ILC1s produce high amounts of TNFα while NK cells mainly produce IFNγ. Although TNFα was first discovered as a rapid inducer of necrosis in tumor cells, it was later shown to have contextual roles in cancer (57). In HCC, TNFα is known to promote carcinogenesis *via* the activation of hepatic progenitor cells (58). Thus, the conversion of NK cells into ILC1-like cells likely favors the development of HCC.

Although, this demonstration was made in a fibrosarcoma model, Gao et al. were able to reconstitute NK, intILC1s and ILC1s in the livers of MCA1956-injected Rag γc-/- mice after intravenous injection of TGF-β-responsive splenic NK cells. In the liver, TGF-β is known to promote fibrosis and higher concentrations are found in HCC patients-derived supernatants (59). This suggests that NK conversion to intILC1s might take place in HCC and impact its progression.

### ILC2s

ILC2s represent less than 5% of all ILCs in the liver at homeostasis and have been poorly studied in the context of liver diseases. However, it was demonstrated that they play a major role in liver fibrosis (60, 61). Their frequency is increased in fibrotic livers from mice and humans. In mice, the accumulation and activation of liver ILC2s was mediated through the IL-33/ST2 axis. Activated ILC2s start producing IL-13 which binds to its receptor IL-13Rα on hepatic stellate cells (HSCs) and triggers their differentiation from “quiescent” to “activated” cells leading to collagen deposition (60) (**Figure 1B**). In patients, the number of ILC2s is correlated to the severity of the fibrosis (61). Given that liver fibrotic patients are at high risk of developing cancer, this suggests a detrimental role for ILC2s in the HCC development.

This was confirmed in a recent study by Xu et al. where the abundance of ILC2s in the tumor area of HCC patients liver is correlated with poor prognosis (62). When looking closely at the phenotype, they identified a subset of ILC2s, enriched in the tumor tissue but absent in the non-tumoral area, lacking the expression of killer cell lectin-like receptor subfamily G member 1 (KLRG1), a known marker for mature and activated ILC2s. KLRG1 interacts with cadherins and particularly E-cadherin. Co-cultures of murine hepatic ILC2s with Hepa1-6 cells resulted in significant decrease in KLRG1 levels. Hepa1-6 cells do not express E-cadherin but overexpression of its *Cdh1* gene maintained KLRG1 expression in ILC2s (62). These experiments suggest that the loss of KLRG1 on ILC2s in HCC can be accounted for by a decrease in E-cadherin expression at the surface of hepatocytes. KLRG1^-^ ILC2s isolated from HCC patients produced significant higher levels of IL-13 and CXCL2 and CXCL8, two chemokines known to recruit neutrophils. All those observations were confirmed in a c-Myc/NRas-induced murine HCC model where hepatic KLRG1^-^ ILC2s produced CXCL2 and IL-13 at higher levels. *Klrg1* knockout (ILC2-CRISPR-KLRG1) and *Klrg1*-overexpressing (ILC2-PCDH-KLRG1) murine ILC2s were generated to assess its specific role in HCC. *Klrg1* overexpression in ILC2s led to a decrease in *Cxcl2* and *Il-13* mRNA levels whereas *Klrg1* knockout enhanced their expression. Neutrophils recruitment was also increased with the use of conditioned medium of *Klrg1*-deficient ILC2s in a chemotaxis assay (62). An immunosuppressive profile was induced in recruited neutrophils *via* the upregulation of *Arg1*, coding for Arginase 1. This increase was likely mediated by IL-13 produced by ILC2s as CXCL2 deficiency did not affect *Arg1* expression. Altogether, these results showed that ILC2s, which downregulate KLRG1 in the HCC tumor microenvironment, promote HCC *via* CXCL2-dependent neutrophil recruitment and IL-13-driven immunosuppression (**Figure 1B**).

### ILC3s

Although rare in the liver, ILC3s might have substantial effects on the development of HCC. ILC3s are subdivided into NCR^+^ ILC3s that represent the main source of intrahepatic IL-22 and NCR^-^ ILC3s that largely produce IL-17A (29, 30). The pro-tumorigenic role of IL-17A was studied. To note, IL-17A can also be secreted by Th17 T cells and γδ T cells. A significant reduction in tumor growth was observed in IL-17A-deficient mice in heterotopic models of HCC. Conversely, intravenous injection of recombinant IL-17A led to an increase in tumor volume. Mechanistically, IL17-A suppresses the cytolytic activity and cytokine production of CD8^+^ T cells and promotes the recruitment of MDSCs through the CXCL5/CCR2 axis (63). Although the Vγ4 γδ T cells were responsible for the IL-17A secretion, this study marked the importance of this cytokine in HCC development. In another study, the same team showed that in mice, IL-17A-producing NCR^-^ ILC3s also participate to the progression of HCC in an IL-23-dependent manner by directly regulating CD8^+^ T cell proliferation and enhancing their apoptosis (**Figure 1C**). However, Vγ4 γδ T cells and ILC3s do not operate at the same stage of the disease. In IL-23-stimulated mice, ILC3s are the main producers of IL-17A at 1 week after intravenous injection of Hepa1.6 cells while CD4^+^, CD8^+^ and γδ T cells become the main IL-17A-producing populations from the second week after injection (64). These results were confirmed in an orthotopic surgical HCC model. Thus, hepatic ILC3s are early responders through the IL-23/IL-17A axis in the context of HCC and seem to promote the progression of the disease.

Interestingly, in a study on human fibrotic livers, a decrease in NKp44^-^ ILC3s was observed at the most severe stages of the disease. Since cirrhotic patients have the greatest risk at developing HCC, this observation questions the actual impact NCR^-^ ILC3s might have on HCC (65).

NCR^+^ ILC3s are even rarer in the liver than their NCR^-^ counterparts. Their implication in HCC has not been studied yet even though IL-22 has been presented with controversial roles in hepatocytes, either promoting their regeneration (model of Con-A induced hepatitis) (66) or their proliferation in the diethyl-nitrosamine (DEN)-induced mouse HCC model (66–68). In both cases, the effect is mediated by the activation of the STAT3 pathway. Thus, NCR^+^ ILC3s could potentially participate to HCC aggravation *via* their secretion of IL-22.

## Signaling Pathways Involved In HCC: Impact On ILCs

During HCC, there is remodeling and reactivation of numerous signaling pathways. We selected the signaling pathways that are the most conserved in development and could then occur both on hepatocytes and immune subsets such as ILCs.

### Notch Pathway

The Notch signaling is one of the most evolutionary conserved pathways. It controls cell fate decision, development and function of numerous cell types including immune cells, and it enables direct cell-to-cell communication (69). First studied in *Drosophila melanogaster*, the Notch pathway displays more complexity in mammals with four heterodimeric receptors (Notch 1-4) that can all bind to five ligands (Jagged 1 and 2, and Delta like ligands 1, 3, 4) with variables affinities (70). When a receptor engages one of its ligands, the extracellular domain is cleaved by ADAM metalloproteases while the Notch intracellular domain (NICD) undergoes serial proteolytic cleavages by the gamma secretase which leads to its translocation to the nucleus of the responding cell (69–71). There, it binds to the transcription factor like recombination signal binding protein for immunoglobulin Jk region (RBP-Jk). Histone acetyltransferases (HAc) among other members of the MAML family are recruited leading to the formation of the NICD/MAML/RBP-Jk activation complex. The latter is responsible for the signaling cascade that enables the transcription of Notch target genes (69–71).

Although Notch is mostly known for its roles in embryonic development and adult tissue homeostasis, it has also been shown to be involved in cancer with pro- or anti-tumorigeneric effects. In HCC, numerous studies have underlined its carcinogenic action. Overexpression of Notch correlated with a decreased overall survival and aberrant expression is found in 30% of HCC patients (72). Notch 3 can regulate the activation of HSCs in the context of fibrosis and its overexpression in HSC leads to an increase in the expression of α-SMA and collagen I (73). Notch signaling can also be activated through IL-6/STAT3 axis leading to the acquisition of stem-like characteristics by HCC cells (74). However, Notch impact on HCC through its activation of immune cells has been poorly studied.

**Figure 2:**
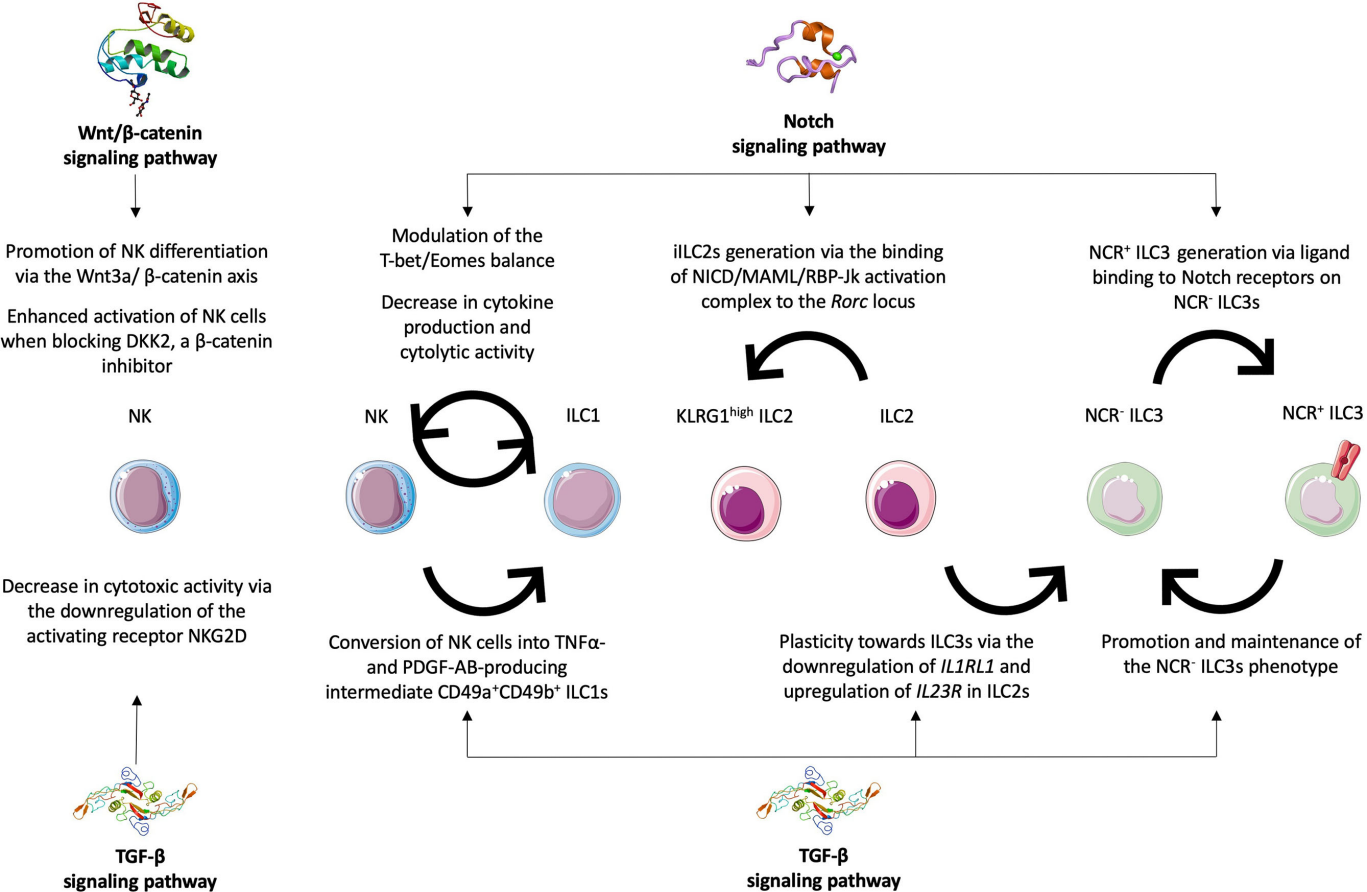
Roles of Notch, TGF-β and Wnt/β-catenin signaling pathways in ILC plasticity and functions. The Notch signaling pathway was implicated in balancing the T-bet/Eomes expression as well as decreasing cytokine production and cytolytic activities in NK cells and ILC1s thus impacting their phenotypes and functions. The Notch signaling is required for the generation of iILC2s, the KLRG1^high^ subset, *via* the binding of the NICD/MAML/RBP-Jk activation complex to the *Rorc* locus. It is also required for the transdifferentiation of NCR^-^ ILC3s into their NCR^+^ counterparts. The TGF-β signaling acts antagonistically and favors the production of NCR^-^ ILC3s either from NCR^+^ ILC3s or from ILC2s *via* the downregulation of *IL1RL1* and the upregulation of *IL23R*. It was also shown to promote the transdifferentiation of NK cells into intermediate CD49a^+^ CD49b^+^ ILC1s in different tumor models. The TGF-β signaling is also involved in decreased NK cytotoxicity by downregulating the activating receptor NKG2D. The Wnt/β-catenin signaling pathway was mostly shown to have an impact on NK cells by promoting their differentiation and functional activation.

Notch signaling is not directly implicated in NK development as its abrogation does not prevent the formation of mature NK cells in the bone marrow (75, 76). Its role in ILC1 remains unknown although Notch is not required for early ILC commitment (77). However, Notch can influence NK and ILC1 functions by modulating the expression of the transcription factor T-bet, necessary for both NK and ILC1 maturation and activation (**Figure 2**). Indeed, it was shown that around half the hepatic ILC1 and NK cells express Notch1 and/or Notch2 (78). Using models such as the IL7R^Cre^ RBP-Jk^flox^ mouse strain, where the Notch pathway is defective in lymphoid cells and their progenitors, the deficiency in RBP-Jk was directly correlated to a shift in the T-bet/Eomes expression balance with the first being decreased and the second increased. Moreover, the levels of CD49a were decreased in RBP-Jk-deficient ILC1s and this was specifically linked to Notch1 signaling as hepatic ILC1s from Il7R^Cre^ Notch2^flox^ mice were not affected (78). Liver NK and ILC1s were also shown to have enhanced cytokine production and cytolytic activity with better control of initial stage of hepatic tumor in the absence of Notch signaling in these heterotopic models (78) (**Figure 2**). By regulating their functions, Notch signaling pathway could thus be considered as one of the factors influencing NK cell exhaustion and tumor progression in HCC.

Notch was proposed as essential for adult ILC2 development from both murine bone marrow CLPs and human progenitors (79). But Notch is also involved in ILC2 plasticity towards ILC3-like cells. ILC2s can be divided into resident ILC2s and inflammatory ILC2s (iILC2s), defined as the KLRG1^high^ circulating subset. ILC2s produce Th2 cytokines while iILC2s can express low amount of RORγt, in addition to high levels of GATA-3, to produce IL-17A along with Th2 cytokines (80). iILC2s have been shown to be major inducers in airway inflammation after challenging mice with house dust mite (81). *In vivo* injections of antibodies targeting Notch1 and/or Notch2 showed that Notch could favor the plasticity towards RORγt-expressing ILC2s. Furthermore, the NICD/MAML/RBP-Jk activation complex can directly bind to the *Rorc* locus which drives the ILC3 differentiation (81) (**Figure 2**). Thus, Notch signaling is possibly required for iILC2 generation. Since KLRG1^-^ ILC2s were implicated in HCC progression, the generation of KLRG1^high^ ILC2s could appear as beneficial. However, iILC2 role has not been clearly defined in HCC making it difficult to conclude.

In the case of the ILC3s, Notch was shown as essential for the differentiation of NCR^-^ ILC3s into NCR^+^ ILC3s (**Figure 2**). This process of maturation is driven by T-bet and Notch2 signaling (36). Indeed, in mice lacking RBP-Jk or Notch2 expression, the numbers of intestinal NKp46^+^ ILC3s were dramatically decreased (36, 82). Moreover, *in vitro* culture of NKp46^-^ ILC3s on Notch ligand expressing OP9-DL1 stromal cells led to the generation of NKp46^+^ ILC3s. However, when cultured on OP9 stromal cells alone, the same cells were not able to differentiate (83). T-bet deficiency also prevented NKp46^-^ ILC3s from giving rise to NKp46^+^ ILC3s regardless of the stromal cells used (83). Notch is thus required for the generation of IL-22 producing NCR^+^ ILC3s. Use of *Ncr1* fate mapping mice revealed a heterogeneity among NCR^-^ ILC3 precursors with some of them having transitionally expressed *Ncr1*. *In vitro* culture for 9 days of NCR^+^ ILC3s on OP9 stromal cells did lead to 40% of the cells losing their expression of Ncr1 (83). Thus, they concluded that Notch is not only necessary for the production of NCR^+^ ILC3s but also to maintain the expression of *Ncr1* and consequently maintain their identity.

### TGF-β Pathway

Transforming growth factor (TGF)-β is a pleiotropic cytokine involved in many biological processes from cell fate and differentiation to proliferation, migration and apoptosis. In the canonical TGF-β signaling pathway, a molecule of active TGF-β engages the monomeric type II receptor (TGF-βRII) which in turn recruits the serine/threonine kinase type I receptor (TGF-βRI) and triggers its cross-phosphorylation (84). The heteromeric complex that is formed is able to phosphorylate SMAD2 and SMAD3 proteins. This enables them to form a transcriptional complex with SMAD4 that regulates the expression of target genes by binding to their regulatory regions. Non-canonical signaling pathways involving other factors exist and participate to the diversity of TGF-β biological roles. Notably, the TGF-β receptor complex can also transduce its signal through mitogen-activated protein kinases (MAPKs), phosphatidylinositide-3 (PI-3) kinase, Rho family GTPases or TNF receptor-associated factor 4/6 (TRAF4/6). Some of their downstream molecules can however interact with the SMAD proteins such as the collaboration of JNK/p38/ERK with SMADs in the regulation of proliferation and cell death (85, 86). TGF-β-mediated activation of TRAF proteins can induce the NF-KB pathway, involved in many inflammatory responses (87, 88). Thus, TGF-β can signal through a great diversity of pathways enabling it to have a substantial variety of roles.

However, TGF-β is most known for its immunosuppressive properties which in cancers can lead to tumor immune-evasion and disease aggravation. In HCC, TGF-β is highly expressed in the liver but its role in tumor development and progression is stage-dependent (89). In the early onset of the disease, TGF-β tends to have an anti-tumor role with the restriction of hepatocyte proliferation. But TGF-β is also strongly associated with liver fibrosis/cirrhosis (90, 91). It is massively produced by HSCs and liver sinusoidal endothelial cells and induces the activation of hepatic Treg cells (92, 93). TGF-β also promotes the generation of pro-inflammatory Th17 cells through the activation of SMAD2/3 in naïve CD4^+^ T cells (94). Moreover, it was shown to induce a shift from M1 towards M2 macrophages and to inhibit the cytotoxic activity of CD8^+^ T cells *via* the suppression of their IFNγ secretion (95, 96). However, its impact on ILC populations in HCC is still not known.

For NK cells to display anti-tumoral role, recognition and targeting of tumor cells are essential. One of the main mechanisms by which NK cells recognize cancerous cells is the engagement of their activating receptors with the ligands expressed at the surface of target cells. The most studied activating receptor is NKG2D that can bind to several ligands (MICA, MICB, ULBP1-6), usually highly upregulated by tumor cells (97, 98). The role of TGF-β in decreasing NKG2D expression on NK cells has been widely studied. Some studies showed that in cancer patients, plasma levels of TGF-β were negatively correlated with the level of expression of NKG2D on circulating NK cells (99, 100). *In vitro* incubation of NK cells with plasma from patients led to a downregulation of NKG2D while the expression was restored with the addition of neutralizing anti-TGF-β antibody (99). Incubation with recombinant TGF-β1 also specifically reduced NKG2D surface expression impairing NK cell cytotoxicity (101). Other studies showed that TGF-β was also responsible for a decrease in NKp30, DNAM-1, granzyme A and perforin expressions and that this was mediated by SMAD2/3 signaling (102, 103). Treatment with the TGFβRI kinase inhibitor Galunisertib (104) or neutralizing anti-TGF-β1 antibody (105) restored the expression of these specific transcripts. Altogether, these results show that TGF-β-mediated NKG2D downregulation participates to the inhibition of NK cytotoxicity in cancer (**Figure 2**).

However, TGF-β has recently emerged as a driver in ILC plasticity. Several studies revealed the implication of TGF-β in multiple cancer diseases into generating a pro-angiogenic NK population (106–108). CD56^+^ NK cells from patients with non–small cell lung cancer (NSCLC) or squamous cell carcinoma (SCC) showed enhanced production of vascular endothelial growth factor (VEGF) and placental growth factor (PIGF) (108). *In vitro* culture of peripheral blood CD56^+^ NK cells from healthy donors with TGF-β resulted in the upregulation of VEGF and PIGF highlighting the essential role of this cytokine in polarizing NK cells towards a pro-angiogenic phenotype (108).

In 2017, Gao et al. went further in describing how TGF-β mediated the conversion of NK cells into intILC1s and ILC1-like cells in several cancer models (56) (**Figure 2**). Given that the tumor-infiltrating NK cells produce large amounts of IFNγ which is one of the key cytokines to inhibit tumor growth and that intILC1s and ILC1s mainly produce the pro-angiogenic molecule PDGF-AB and pro-tumorigenic cytokine TNFα (58), TGF-β signaling is considered as prone to favor tumor immune evasion. Additionally, using single-cell RNA sequencing and flow cytometry analysis of liver ILCs from HCC patients, another study revealed the presence in the tumor area of an NK-like population with a mixed NK/ILC1 phenotype. TGF-β mRNA levels were found to be significantly increased in the tumor area (109). These results suggest that TGF-β signaling could be involved in HCC progression by promoting NK conversion into ILC1-like cells. In another study, liver-derived TGF-β was shown to sustain the Eomes^hi^ T-bet^low^ phenotype in human liver NK cells (110). Given that the CD49a^+^ CD49b^-^ cells that might be considered as human counterparts of ILC1s are T-bet^+^Eomes^-^, the role of TGF-β in NK conversion to ILC1-like cells remains to be confirmed in patients.

TGF-β was also found to drive the plasticity of ILC2s and ILC3s. Recent studies revealed that human ILC2s cultured in presence of TGF-β, IL-1β and IL-23 can transdifferentiate into IL-17A-producting ILC3-like cells (111, 112). TGF-β is not necessary as *in vitro* culture of ILC2s with IL-1β and IL-23 can lead to IL-17A production. However, when TGF-β is added to the medium, the secretion of IL-17A is dramatically increased while that of IL-5 is decreased. This was explained by the substantial upregulation of *IL23R* in the presence of TGF-β as well as the reduced mRNA expression of *IL1RL1*, the gene coding for the IL-33 receptor ST2 (**Figure 2**). By increasing the response to IL-23 and decreasing the one to IL-33, TGF-β promotes the conversion of ILC2s into ILC3s that produce the pro-inflammatory IL-17A cytokine.

TGF-β was also shown to have a direct action on ILC3s to promote the NCR^-^ phenotype (**Figure 2**). In the same study that revealed an essential role for Notch signaling in NCR^+^ ILC3 generation, TGF-β was shown to act in opposition (83). TGF-β signaling impairs the differentiation of NCR^-^ ILC3s in NCR^+^ ILC3s *in vitro* and *in vivo.* But it can also drive the reverse conversion of NCR^+^ ILC3s into NCR^-^ ILC3s as *in vitro* culture of NCR^+^ ILC3s that express a constitutive active form of TGF-βRI leads to a decreased expression of *Ncr1* and decreased numbers of NCR^+^ cells (83). Given that NCR^-^ ILC3s secrete higher amounts of IL-17A and were implicated in HCC progression, we can speculate that TGF-β may participate to liver tumorigenesis by mediating ILC2 and ILC3 plasticity.

### Wnt/β-catenin Pathway

The Wnt/β catenin signaling pathway is a highly conserved pathway that controls embryonic development, cell proliferation, differentiation and fate determination (113). In mammals, 19 genes coding for different Wnt proteins are expressed (114, 115). The latter are lipid-modified in the endoplasmic reticulum and are then transported from the Golgi to the cell membrane thanks to the chaperone Wntless (116). Once secreted, the Wnt proteins can interact with the Frizzled (FZD) receptor at the surface of the responding cell. The FZD are associated with coreceptors, either LRP5/6 or the ROR/RYK complex. Binding to the FZD/ROR/RYK usually leads to the activation of the Wnt/β-catenin-independent pathway while interaction with FZD/LRP5/6 usually results in the activation of the canonical Wnt/β-catenin signaling pathway (117). The β-catenin is a protein found in the cytoplasm of cells. In the absence of interaction between Wnt and FZD, it is ubiquitinated by the β-catenin destruction complex, composed of the casein kinase 1 (CK1), the GSK-3β, the adenomatous polyposis coli (APC) and AXIN1, leading to its subsequent degradation by the proteasome (113, 114, 118). However, upon binding of Wnt to FZD, the scaffolding DVL protein is recruited to the FZD intracellular domain which leads to the inhibition of GSK-3β (119). The β-catenin is thus stabilized and can translocate to the nucleus where it promotes the transcription of target genes (120).

Although Wnt/β-catenin signaling is known to take part in many essential biological processes, its dysregulation was shown to be involved in HCC development (121, 122). One study also revealed how in HCC, tumor-derived Wnt ligands polarize tumor-associated macrophages (TAM) towards an M2 phenotype contributing to tumor progression (123).

The Wnt/β-catenin pathway was shown to be involved in NK cell differentiation. Exposure of human thymic CD34^+^CD1a^-^ progenitors to Wnt3a that signals through the β-catenin led to an increased production of NK cells compared to untreated samples (124) (**Figure 2**). Moreover, the blockade of Wnt signaling *via* DKK1, a known inhibitor of Wnt/β-catenin, in human CD34^+^ hematopoietic progenitors led to a significant decrease in NK cell generation (125). Another study showed that β-catenin-deficient mice have decreased NK cell numbers with an action of β-catenin on the expression of the antiapoptotic protein Bcl2 (126). However, the Wnt/β-catenin pathway could also be implicated in NK cell function and cytotoxic activity. In one study, blockade of DKK2, another inhibitor of the β-catenin pathway, led to an enhanced activation of NK cells (**Figure 2**). Using a colorectal cancer mouse model, they injected 5F8, a molecule specifically preventing the binding of DKK2 to LRP5. It resulted into a decrease in the numbers of cancerous intestinal polyps. This suggests that DKK2 promotes tumor progression in this model. And this effect could be partly mediated by NK cells whose cytotoxic activity is enhanced in the absence of DKK2. Indeed, administration of 5F8 led to significant increases in *Gzmb*, *CD69*, *IFNγ* and *NKp46* gene expression (127). Although set in the colorectal cancer model, this study underlines the importance of Wnt/β-catenin signaling pathway in NK-driven anti-tumoral response. It can thus be hypothesized that Wtn-β-catenin could also regulate NK cell activation in HCC.

Implication of Wnt proteins in the differentiation and/or function of helper ILCs has not been studied yet. Several studies underpinned the role of the transcription factor TCF-1 in ILC2 and ILC3 differentiation. However, TCF-1 is not only a Wnt/β-catenin signaling target gene but is also a downstream target gene of the Notch pathway. One study showed that β-catenin-deficient hematopoietic progenitors, Lin^−^Sca-1^+^c-Kit^+^ (LSK) cells, can develop normally *in vivo* into ILC2 while Notch-inhibited LSK cells fail to produce ILC2s. This suggests that Wnt-β-catenin pathway might not be involved in ILC2 differentiation as the Notch signaling (128).

## Conclusion

ILCs are emergent actors in the field of cancer research. Here, we have summarized their roles in HCC and underlined the potential impact the Notch, TGF-β and Wnt/β-catenin signaling pathways might have on their response. The cytotoxic NK cells remain to this day the most studied cells however, helper liver ILCs are being increasingly scrutinized as their tissue residency characteristics position them as chronically exposed to this specific tolerogenic environment.

Another highlight of ILCs is their ability to transdifferentiate from one subset to another. Although this plasticity is mostly driven by the presence of specific interleukins, Notch signaling and TGF-β pathways are major actors in this process and can even harbor antagonist roles. Wnt/β-catenin signaling pathway impact has been poorly investigated but one can expect future research to uncover its role in ILCs in cancer and most specifically in HCC.

Further studies are needed to clarify the roles of these signaling pathways in immune cells, and in particular ILCs, in HCC as many studies have been performed in other cancer models. This will provide new insights into the molecular mechanistic underlying the response of ILCs and subsequently allowing new immunotherapeutic strategies to emerge in order to specifically target these signaling pathways in ILCs.

## Author Contributions

EB designed and prepared the manuscript and the figures. RG gave guidance on the outline and revised the manuscript. All authors contributed to the article and approved the submitted version.

## Funding

This work has been supported by Institut Pasteur, Université de Paris, Institut National de la santé et de la recherche Médicale (INSERM) and by grants from the French Government (National Research Agency, ANR), ANR project NASHILCCD8 (#18-CE15-0024-01).

## Conflict of Interest

The authors declare that the research was conducted in the absence of any commercial or financial relationships that could be construed as a potential conflict of interest.

## Publisher’s Note

All claims expressed in this article are solely those of the authors and do not necessarily represent those of their affiliated organizations, or those of the publisher, the editors and the reviewers. Any product that may be evaluated in this article, or claim that may be made by its manufacturer, is not guaranteed or endorsed by the publisher.
